# Evaluation of Adipokine Status and Leptin Receptor Gene Polymorphism in Patients with Severe Asthma

**DOI:** 10.3390/diagnostics15091154

**Published:** 2025-05-01

**Authors:** Saule Maimysheva, Lyudmila Karazhanova, Andrey Orekhov, Assel Chinybayeva, Bolat Ashirov

**Affiliations:** 1 Department of Internal Medicine, Semey Medical University, Semey 071400, Kazakhstan; maimysheva@mail.ru (S.M.); kar_ludmila@bk.ru (L.K.); 2 Cardiology Department, Astana Medical University, Astana 010000, Kazakhstan; chena@bk.ru; 3 Department of Internal Medicine and Cardiology, South Kazakhstan Medical Academy, Shymkent 160019, Kazakhstan; bolat.baja@mail.ru

**Keywords:** asthma, metabolic syndrome, obesity, interleukine, leptin receptor, polymorphism

## Abstract

**Background**: Severe and difficult-to-control asthma occurs in 3–10% of patients in developed countries. The aim of our study was to investigate the association of the prognostic role of leptin and adiponectin, as well as the leptin receptor gene polymorphism Gln223Arg, in patients with difficult-to-control and severe asthma. **Methods**: The present study included 200 patients with asthma hospitalized in the Department of Pulmonology between January 2018 and December 2021. In all patients, in addition to routine clinical investigations, adiponectin, leptin and their ratio were analyzed, as well as levels of pro-inflammatory cytokines (IL-6, IL-8 and TNF-alpha). External respiratory function was also assessed. LEPR Gln223Arg single-nucleotide polymorphisms were genotyped by real-time PCR method. **Results**: Patients were randomized into two groups, depending on the severity of asthma: an uncontrolled asthma group and a controlled asthma group, according to the GINA criteria. Among patients with uncontrolled asthma, 101 subjects (74.3%) had metabolic syndrome (*p* < 0.001). There was an inverse association of the adiponectin/leptin ratio with the eosinophil count (B = −0.305, *p* < 0.001), IL-6 (B = −0.026, *p* < 0.001), IL-8 (B = −0.062, *p* < 0.001) and TNF-alpha (B = −0.047, *p* < 0.001) and a direct correlation with the level of FEV1 (B = 0.121, *p* < 0.001) and FVC (B = 0.104, *p* < 0.001). A probable association of homozygous A/A allele with increased risk of uncontrolled asthma was shown (*p* = 0.007). **Conclusions**: Leptin receptor polymorphism with A/A genotype may be associated with a higher probability of developing severe and difficult-to-control asthma.

## 1. Introduction

Asthma is a heterogeneous disease associated with inflammation of the airways and is one of the most common chronic non-communicable diseases [1]. According to the published report of GBD 2019 Diseases and Injuries Collaborators, the prevalence of asthma in the world continues to grow, amounting to 262.41 million cases in 2019 (95% CI 224.05–309.45) [2,3]. Currently, certain success has been achieved in the treatment of asthma. The approach to the treatment of asthma proposed by GINA experts, based on step-by-step pharmacological therapy with correction of risk factors, improves control of the disease symptoms [1]. However, there remains a portion of patients with severe or “difficult-to-control” asthma, the prevalence of which is about 3–10% in developed countries [4,5].

The disease’s many distinct clinical phenotypes pose significant challenges to effectively controlling asthma [6]. In addition, the discovery of different endotypes [7] shows that the pathogenetic mechanisms of asthma are not all the same. One of the phenotypes is the association between asthma and obesity. According to modern concepts, being overweight directly causes and keeps metabolic inflammation going. This is because being overweight causes a metabolic imbalance and persistent overproduction of proinflammatory mediators [8]. Studies indicate that obesity is one of the frequent comorbidities in patients with asthma [9]. Moreover, obesity and overweight may be significant risk factors for poor control and a severe course of asthma. Thus, the TENOR II study indicated that the prevalence of obesity in patients with severe and difficult-to-control asthma is 25.5%, while in patients with optimal symptom control it is 17.1% [10]. One of the factors linking obesity and metabolic syndrome is insulin resistance (IR), the presence of which may be one of the factors predisposing to the development of asthma [11]. So, it has been shown that IR causes a number of structural and functional problems, such as a progressive drop in FEV1 and FVC and less response to treatment [12]. To assess the severity of metabolic disorders, the study of adipokine status may be recommended as a marker of adipose tissue dysfunction. Studies have shown that patients with asthma, especially its severe form, are characterized by higher leptin levels compared to patients without asthma [13]. Furthermore, studies have found a positive correlation between leptin and the severity of asthma, a relationship that adiponectin did not show [14]. The adiponectin/leptin ratio is a new IR marker that has been shown to be useful in studies of people with cardiovascular disease [15], but there is not much evidence that it can be used in people with asthma. We are still looking into the part that the leptin receptor gene Gln223Arg plays in the development of asthma and how it affects controlling symptoms [16,17].

The aim of our study was to investigate the association of the prognostic role of leptin and adiponectin, as well as the leptin receptor gene polymorphism Gln223Arg, in patients with difficult-to-control and severe asthma.

## 2. Materials and Methods

### 2.1. Characteristics of the Study Groups

The present study included 200 patients with asthma hospitalized in the Pulmonology Department of the Emergency Hospital in Semey, Republic of Kazakhstan, between January 2018 and December 2021. Inclusion criteria for the study: asthma, diagnosis was established according to national and international criteria [18]; age older than 18 years. All patients were treated according to GINA stage 4–5. Exclusion criteria from the study: smoking, presence of chronic obstructive pulmonary disease, active infectious disease, cancer. Patients were divided into 2 groups depending on the degree of asthma control, which was assessed using the AST test (Table 1).

### 2.2. Collection of Clinical and Laboratory Data

All patients underwent clinical blood tests, biochemical blood analysis with determination of lipid spectrum and fasting blood glucose level. Clinical analysis of sputum was also performed. Blood concentrations of leptin, adiponectin, IL 6, IL 8 and TNF-α were studied using available commercial ELISA kits according to the manufacturers’ official instructions. Spirometry was performed on admission of all patients to the Pulmonology Department by trained staff with subsequent interpretation of the results by the investigator. The study was performed on a BTL-08 Spiro Pro (UK), and the final report included only the results of tests performed according to the recommendations of the American Thoracic Society and European Respiratory Society [19]. The following parameters were evaluated during the study: FEV1 and FVC.

#### Leptin Receptor Gene Polymorphism Gln223Arg

Venous blood drawn into vacuum tubes with K2/K3 EDTA was used for the study. Genomic DNA was isolated from blood using the Gene JET Thermo Scientific (Vilnius, Lithuania) Gene JET kit. The isolated DNA was frozen and stored at −20 °C. We performed a quantitative and qualitative evaluation of the isolated DNA using a nanospectrophotometer (NanofotometerP30, IMPLEN, Germany) and electrophoretically in an agarose gel. We used a CFX 96 (BioRad, Hercules, CA, USA) for real-time PCR on 96 samples (25 µL) with ready-made primer mixtures and TaqMan probes, along with TaqMan Genotyping Mastermix reagent (reagents produced by Life Technologies, Carlsbad, California, USA). The amplification program for SNP Gln223Arg (rs1137101) included pre-denaturation at 95 °C for 3 min, followed by 40 cycles of 95 °C for 15 s and 65 °C for 40 s. For SNP Gln223Arg (rs1137101), the amplification program included a pre-denaturation step of 93 °C for 1 min, followed by 35 cycles of 93 °C for 10 s, 64 °C for 10 s, and 72 °C for 20 s.

### 2.3. Ethical Approval Details

All patients confirmed their consent to participate in the study and the results to be published. The study was approved by the Local Ethical Committee of NC JSC “Semey Medical University” (decision No. 11 dated 27 September 2017).

### 2.4. Statistical Analysis

Statistical processing of data was performed using the SPSS 20.0 and SNPStat version 2.2.1. Quantitative indicators were evaluated for conformity to normal distribution using the Kolmogorov–Smirnov criterion. Quantitative indices having normal distribution were described using arithmetic mean (M) and standard deviations (SD), 95% confidence interval (95% CI) limits; Student’s criterion for independent samples was used for comparison. In the case of non-normal distribution, quantitative data were described using median (Me) and lower and upper quartiles (Q1–Q3). Comparison between two groups for a quantitative measure whose distribution differed from normal was performed using the Mann–Whitney U-criterion. Categorical data were described with absolute values and percentages, and Pearson’s χ^2^ was used to identify the relationship between nominal variables. The direction and closeness of the correlation relationship between two quantitative indicators were assessed using Spearman’s rank correlation coefficient (in the case of distribution of indicators other than normal). Single-factor regression analysis was also performed to determine the influence of factors affecting the risk of severe asthma. The χ^2^ test was used to determine whether the genotype frequency distribution conformed to the Hardy–Weinberg equilibrium (HWE) (*p* > 0.05 was indicative of conformity to the Hardy–Weinberg equilibrium).

## 3. Results

All patients were assessed for the presence of metabolic syndrome according to the accepted criteria (WHO). In this case, 112 (56%) patients had verified metabolic syndrome, and 88 (44%) individuals did not have these criteria. Of the patients with uncontrolled BA, 101 (74.3%) had metabolic syndrome, while only 11 (17.2%) of those with controlled BA did. These differences were statistically significant (*p* < 0.001).

Adipokine status was studied in all patients included in the study (Table 1). The median leptin level was 15.24 (5.42–21.67) ng/mL and the adinonectin level was 6.5 (5.38–14.95) ng/mL, and no significant differences in adipokine levels were found between men and women (*p* = 0.33 for adiponectin and *p* = 0.448 for leptin). Significant differences were found in leptin and adiponectin levels among patients with uncontrolled and controlled asthma (*p* < 0.001 for leptin and adiponectin).

Table 2 presents the results of linear regression analysis to assess the relationship between the adiponectin/leptin (A/L) ratio and some clinical and laboratory characteristics of patients with asthma. Thus, an inverse relationship between A/L and the number of eosinophils in peripheral blood, as well as with proinflammatory cytokines such as IL-6, IL-8 and TNFα, was established. A direct relationship, manifested in an increase in both parameters, was established with the studied indices of external respiratory function (FEV1, FVC).

**Figure 1:**
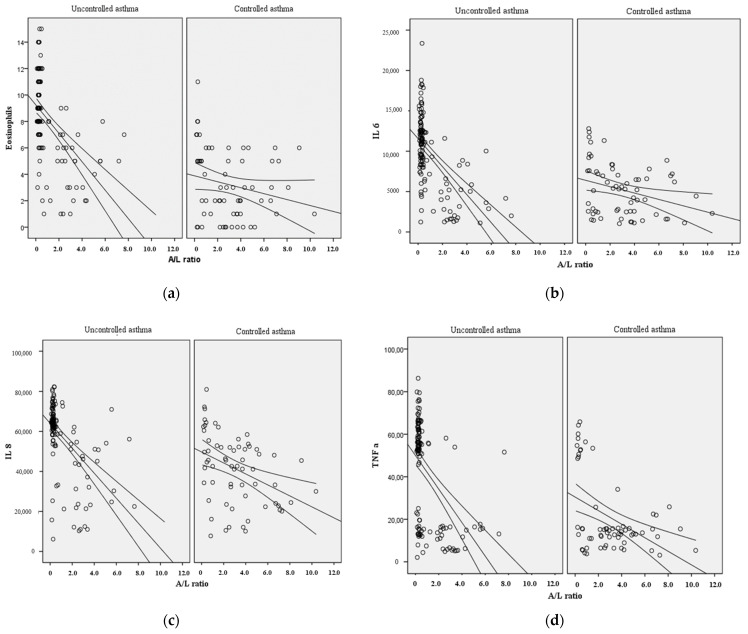
Scatterograms of A/L ratio dependence on spirometry parameters with level of eosinophils (**a**) and proinflammatory cytokines (**b**–**d**).

The studied parameters were further evaluated depending on the degree of asthma control. It was found that an increase in the number of eosinophils in the peripheral blood should be expected when the A/L ratio decreases (Figure 1a). In the obtained model of patients with uncontrolled/severe asthma, the coefficient of determination R2 Nagelkerke was 0.273; i.e., the model explains 27.3% of the variance of the studied ratio, which corresponds to an appreciable closeness of relationship (r = 0.546). Among patients with controlled asthma, the R2 Nagelkerke coefficient was 0.045; i.e., the A/L ratio explained 4.5% of the variability in eosinophil levels, having a weak linear relationship (r = 0.213).

The relationship between the A/L ratio and proinflammatory cytokines IL-6 (Figure 1b), IL-8 (Figure 1c) and TNFα (Figure 1d) playing a pathogenetic role both in patients with asthma and in patients with abdominal obesity was also studied. Thus, it was found that in an uncontrolled and severe course of asthma it is to be expected that the decrease in the A/L ratio leads to an increase in the studied cytokines. The R2 Nagelkerke coefficient was 0.248 for TNFα and 0.339 and 0.299 for IL-6 and IL-8, respectively. As a result, the A/L ratio was demonstrated to explain 24.8%, 33.9%, and 29.9% of the variability of TNFα, IL-6, and IL-8 with a moderate linear relationship. At the same time, among patients with controlled asthma, the coefficients of determination (R2) were 0.194, 0.093 and 0.152 for TNFα, IL-6 and IL-8, showing a weak dependence (r = 0.39 and r = 0.3050 for IL-6 and IL-8) and a moderate dependence (r = 0.44) for TNFα.

**Figure 2:**
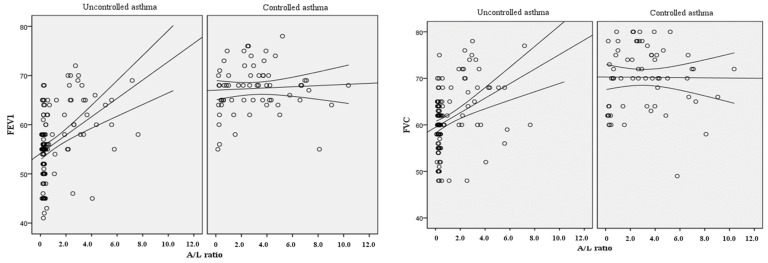
Scatterograms of A/L ratio dependence on spirometry parameters.

The dependence of the A/L ratio on some parameters of external respiratory function was also evaluated (Figure 2). Thus, it was found that an increase in respiratory volumes is also observed with increasing A/L ratio. It was shown that among patients with uncontrolled or severe asthma, the coefficient of determination (R2 Nagelkerke) for FEV1 was 0.173, and for FVC 0.133, thus showing that the obtained model with IR estimation by A/L ratio explains 17.3% and 13.3% of the variability of FEV1 and FVC, respectively. The obtained models had a weak linear relationship (r = 0.416 r = 0.364 for FEV1 and FVC). At the same time, among patients with controlled asthma, no correlation between the A/L ratio and external respiration parameters was found. Thus, the coefficient of determination was 0 and 0.003 for FEV1 and FVC, respectively.

Table 3 presents data on the models evaluating the Gln223Arg polymorphism of the leptin receptor in patients with uncontrolled (*n* = 136) and controlled asthma (*n* = 64). There was a lower chance of getting asthma that cannot be controlled in both codominant and dominant models when A/G and G/G genotypes were present (*p* = 0.024 and *p* = 0.0064 for the two models, respectively). In the log-additive model, it was also found that the G allele may be linked to a lower risk of asthma that is severe and hard to control.

To determine the association between the development of severe and difficult-to-control asthma and the presence of polymorphism in the leptin receptor gene, we performed logistic regression analysis, the results of which are given in Table 4.

## 4. Discussion

For a long time, two main approaches have dominated the literature regarding the determination of pathogenetic links between asthma and abdominal obesity, a key component of MS. According to the first perspective, abdominal obesity causes airway narrowing and increases airway resistance, leading to a reduction in total lung capacity [20]. Additionally, several authors associate the presence of abdominal obesity with airway hyperresponsiveness [21]. Recently, increasing attention has been given to the hypothesis that adipose tissue produces elevated levels of proinflammatory mediators, which in turn enhance airway inflammation, promoting airway hyperresponsiveness and remodeling [22,23]. However, the literature contains limited data on the association between bronchial asthma and metabolic syndrome. At the same time, the prevailing view is that MS is primarily linked to cardiovascular effects, giving rise to the concept of the so-called “metabolic syndemia” [24].

In our study, for the first time in the regional aspect in the Republic of Kazakhstan, the incidence of MS in patients with an uncontrolled course of asthma was found to be 56%. Our data indicate a higher prevalence of MS in the study population compared to the global trend. In a systematic review of eight studies, the prevalence of MS in patients with asthma was found to be 25% (95% CI 13–38%) [23,25]. This high frequency is probably due to the fact that our study included patients with severe asthma, which once again confirms the role of MS in refractoriness to therapy. In addition, previously published data show a significant increase in the prevalence of obesity and MS in Kazakhstan, and experts predict it will grow to 55% by 2030 [26]. This fact requires the expansion of programs for the treatment of obesity and MS in the country and the inclusion of specialists in multidisciplinary programs, including in the treatment of patients with severe asthma.

One of the key factors determining the pathogenetic relationship between severe asthma and MS is insulin resistance (IR). It has been established that adipokines produced by adipose tissue can be divided into two main types: proinflammatory, including leptin and resistin, and anti-inflammatory, primarily adiponectin. Leptin, increasing insulin resistance and increasing triglyceride lipolysis, causes the development of hyperinsulinemia, thus supporting systemic low-intensity inflammation [27]. Hyperinsulinemia, in turn, increases acetylcholine concentration and potentiates the activity of parasympathetic influences on the respiratory tract, resulting in hyperresponsiveness and bronchial remodeling [28]. Adiponectin, on the other hand, has an inhibitory effect on proinflammatory cytokines (IL 6, TNFα) and is considered as a factor that significantly reduces IR. In view of this relationship in patients with metabolic syndrome and obesity, the relationship of these adipokines in patients with asthma was studied. Thus, it was shown that leptin was inversely correlated with the severity of asthma, which was assessed by the level of FEV1 (r = −0.714, *p* = 0.001) and FVC (r = −0.740, *p* = 0.001) [13]. At the same time, no such associations were found with adiponectin levels. Further meta-analysis, including data from 59 studies, also showed that patients with severe asthma are characterized by higher leptin levels compared to controlled asthma (Standard mean difference, SMD 1.638, 95% CI: 0.952–2.323, *p* < 0.0001), which was more significant for Asian patients (SMD: 2.600, 95% CI: 1.854–3.345, *p* < 0.0001) [13]. In addition, meta-regression analysis in this study found that age and gender had no effect on leptin status among patients with severe asthma (coefficient: −0.072, 95% CI: −0.208 to 0.063, *p* = 0.279).

The study of the adiponectin/leptin ratio as a factor of adipose tissue dysfunction is currently of great interest. Thus, it has been shown that this parameter can be a marker of the presence of IR in patients with MS and obesity [29,30]. In addition, its role in cardiometabolic risk assessment has been determined; a level of more than 1.0 corresponds to low risk, while a ratio of less than 0.5 characterizes a significant increase in cardiometabolic risk [31]. At the same time, data on the use of the adiponectin/lipetin ratio in patients with asthma are very limited.

Our study revealed an inverse relationship of the A/L ratio with the number of eosinophils in peripheral blood, as well as with proinflammatory cytokines, such as IL-6, IL-8 and TNFα in patients with asthma. As mentioned above, adiponectin, which has an anti-inflammatory effect, has an inhibitory effect on IL-6 and TNFα. Our work shows that this effect also applies to the A/L ratio. In addition, blood eosinophilia, which is an important pathogenetic factor in asthma and is associated with the level of proinflammatory cytokines, also decreases with increasing A/L ratio (B = −0.305, 95% CI: −0.369–(−0.238), *p* < 0.001) according to our data.

The leptin receptor is made by a single gene called LEPR. The most common variation in this gene is the replacement of glutamine with arginine at position 223 in the protein (Gln223Arg), which affects how the receptor works [32]. There are very limited data in the literature on the effect of polymorphisms in the leptin receptor gene in patients with asthma. One of the few publications that studied these associations among asthma patients in a pediatric population included patients from 6 to 18 years of age [33]. The authors found that leptin polymorphisms may influence serum leptin levels and are probably associated with an increased risk of exacerbations in patients with asthma, but no relationship was identified between LEPR and the severity or control of asthma. In our study, only one LEPR polymorphism, rs12131454 (Glu223Arg), was investigated. So, we show that having the homozygous A/A allele is likely to be linked to a higher risk of getting asthma that gets out of hand (OR = 2.31, *p* = 0.007). Of course, differences in the population of patients included in the study should be taken into account.

Currently, our understanding of the role of leptin receptor gene polymorphism in the development of respiratory pathology is limited. The primary data available focus on the established association between the leptin receptor gene polymorphism rs12131454 (Glu223Arg) and the development of type 2 diabetes mellitus, metabolic syndrome, and obesity [33]. The information presented here does not reliably substantiate a causal relationship between the identified mutation and asthma severity. However, previously published studies have linked asthma, including its difficult-to-control form, to obesity [23]. As mentioned above, abdominal obesity not only exerts a mechanical effect on airway patency but also contributes to chronic low-intensity inflammation stimulated by adipose tissue. This inflammation leads to respiratory tract remodeling due to increased activation of neutrophils and eosinophils, as well as heightened production of pro-inflammatory cytokines. Our study demonstrates an association between pro-inflammatory cytokines and asthma severity. Thus, mutations in the leptin receptor gene, which mediate an increased risk of severe obesity, are likely associated with the development of eosinophilic and neutrophilic endotypes of asthma, with the latter being more prevalent. However, further investigation into the peculiarities of leptin receptor function in cases of mutation is necessary.

### Limitations of the Study

The most significant limitations are the sample size of the study and the inclusion of only one leptin gene polymorphism in the analysis, which does not fully characterize the entire aspect of the clinical phenotype of a patient with severe and difficult-to-control asthma associated with metabolic syndrome.

## 5. Conclusions

With the rise of personalized patient care, it is now necessary to look at the pathogenetic interactions of multiple diseases in a more complete and in-depth way. Syntropy of asthma and metabolic syndrome is becoming increasingly important, given that both conditions can interact and influence each other, leading to worsening symptoms and severe disease course.

The study found that the A/L ratio had a negative relationship with the number of eosinophils in the peripheral blood, proinflammatory cytokines (IL-6, IL-8, and TNF-alpha), and a direct relationship with FEV1 and FVC, which measure how well the lungs are working outside the receptor polymorphism with an A/A genotype, may be linked to a higher risk of getting severe asthma that is difficult to control.

## Figures and Tables

**Table 1:** Clinical characteristics of patients.

Characteristic	All Patients	Group 1 (Uncontrolled Asthma), *n* = 136	Group 2 (Controlled Asthma), *n* = 64	*p*
Age, years	53.5 (39–59.25)	56.0 (46.0–61.0)	41.0 (31.0–55.0)	<0.001 ^b^
Women, *n* (%)	80 (40%)	77 (56.6)	43 (67.2)	0.155 ^c^
Body mass index, kg/m^2^	27.05 (23.1–28.42)	28.01 (26.27–29.32)	22.7 (21.3–24.5)	<0.001 ^b^
Waist circumference, cm	92.5 (85.0–98.0)	95.0 (89.75–98.0)	85.0 (74–86)	<0.001 ^b^
Arterial hypertension, %	87 (43.5)	74 (54.4)	13 (20.8)	<0.001 ^c^
Diabetes mellitus, %	78 (39.0)	70 (51.1)	8 (12.5)	<0.001 ^c^
Eosinophils, cells/µL	84 (52–127.5)	107 (75.8–145)	54 (38–82.75)	<0.001 ^a^
Total cholesterol, mol/L	5.2 (3.3–5.9)	5.65 (4.59–6.1)	3.44 (2.6–4.83)	<0.001 ^b^
Triglycerids, mol/L	2.8 (1.69–3.24)	3.01 (2.62–3.5)	1.78 (1.08–2.21)	<0.001 ^b^
HDL, mol/L	1.2 (0.97–1.64)	1.22 (0.97–1.97)	1.12 (0.98–1.42)	0.241 ^b^
LDL, mol/L	3.18 (2.65–3.74)	3.82 (2.68–3.92)	3.01 (2.11–3.84)	0.136 ^b^
Glucose, mol/L	6.0 (5.1–6.8)	6.4 (7.5–7.33)	5.15 (4.38–5.5)	<0.001 ^b^
Adiponectin	6.5 (5.38–14.95)	6.0 (5.0–11.15)	13.85 (11.1–17.35)	<0.001 ^b^
Leptin	15.24 (5.42–21.67)	18.9 (11.97–22.70)	5.0 (3.41–14.53)	<0.001 ^b^
IgE, IU/mL	107 (85.75–150.0)	109.5 (86.5–141.25)	97.0 (85.75–158.0)	0.648 ^b^
Adiponectin/Leptin	0.4 (0.2–2.62)	0.3 (0.3–0.75)	2.65 (0.75–4.2)	<0.001 ^b^
FEV1, %	58.0 (55.0–65.25)	55.0 (52.0–60.0)	68.0 (65.0–70.0)	<0.001 ^b^
FVC, %	62.0 (60.0–70.0)	60.0 (58.0–65.0)	70.0 (64.0–75.0)	<0.001 ^b^
FEV1/FVC	60.0 (56.0–68.0)	58.0 (55.0–62.0)	68.0 (62.0–72.0)	<0.001 ^b^

Note: ^a^—Parametric criterion: Student *t*-test, M ± SD (mean ± standard deviation); ^b^—Non-parametric criterion: Mann–Whitney U-test, Me(IQR), Q1–Q3; ^c^—χ^2^ Pearson.

**Table 2:** Results of linear regression analysis of the adiponectin/leptin ratio and clinical and laboratory parameters of patients with asthma.

Parameter	B	Standard Error	95% CI	*p*
Eosinophils	−0.305	0.033	−0.369–(−0.238)	<0.001
FEV1	0.121	0.016	0.089–0.152	<0.001
FVC	0.104	0.017	0.07–0.138	<0.001
C-reactive protein	−0.577	0.472	−1.508–0.354	0.223
IgE	0.003	0.003	−0.004–0.01	0.382
TNFα	−0.047	0.005	−0.057–(−0.037)	<0.001
IL-8	−0.062	0.007	−0.075–(−0.049)	<0.001
IL-6	−0.026	0.026	−0.304–(−0.200)	<0.001

**Table 3:** Leptin receptor Gln223Arg gene polymorphism associations in patients with asthma.

Model	Genotype	Severe BA	Non-Severe BA	OR (95% CI)	*p*-Value
Codominant	A/A	57 (41.9%)	40 (62.5%)	1.00	0.024
	A/G	40 (29.4%)	12 (18.8%)	0.43 (0.20–0.92)	
	G/G	39 (28.7%)	12 (18.8%)	0.44 (0.20–0.94)	
Dominant	A/A	57 (41.9%)	40 (62.5%)	1.00	0.0064
	A/G-G/G	79 (58.1%)	24 (37.5%)	0.43 (0.24–0.80)	
Recessive	A/A-A/G	97 (71.3%)	52 (81.2%)	1.00	0.13
	G/G	39 (28.7%)	12 (18.8%)	0.57 (0.28–1.19)	
Overdominant	A/A-G/G	96 (70.6%)	52 (81.2%)	1.00	0.1
	A/G	40 (29.4%)	12 (18.8%)	0.55 (0.27–1.15)	
Log-additive	-	-	-	0.63 (0.43–0.92)	0.014

**Table 4:** Association of leptin receptor Gln223Arg gene polymorphism with asthma severity.

SPN	Severe BA, *n* = 136 (68%)		non-Severe BA, *n* = 64 (32%)		OR	95% CI	*p*
	*n*	%	*n*	%			
A/A	57	40	40	62.5	2.310	1.255–4.251	0.007
A/G	40	29.4	12	18.8	1.806	0.872–3.739	0.112
G/G	39	28.7	12	18.8	0.574	0.277–1.19	0.136

## Data Availability

The data are not publicly available due to confidentiality agreements and privacy concerns but can be accessed upon reasonable request to ensure proper use and adherence to ethical guidelines.

## Abbreviations

The following abbreviations are used in this manuscript:

GINA	Global Initiative for Asthma
MS	Metabolic syndrome
IR	Insulin resistance
FEV1	Forced expiratory volume in 1 s
FVC	Forced vital capacity
HDL	High-density lipoprotein
LDL	Low-density lipoprotein
iGCS	Inhaled glucocorticosteroids
SABA	Short-acting beta-agonists
LABA	Long-acting beta-agonists
IgE	Immunoglobulin E
IL	Interleukin
TNFα	Tumor necrosis factor α
